# Association between maternal mental health-related hospitalisation within the first postnatal year and potentially avoidable hospitalisation among their children in the Northern Territory of Australia, using 16 years of linked administrative data

**DOI:** 10.1007/s00737-026-01774-3

**Published:** 2026-09-30

**Authors:** Demeke Mesfin Belay, Abel Fekadu Dadi, Holger W Unger, Samuel Hailegebreal Gele, Behailu Derseh, Bernard Leckning, Steven Guthridge

**Affiliations:** 1 Centre for Child Development and Education, Menzies School of Health Research, Charles Darwin University, Darwin, NT Australia; 2 College of Health Science, Debre Tabor University, Debre Tabor, Ethiopia; 3 Addis Continental Institute of Public Health, Addis Ababa, Ethiopia; 4 Global and Tropical Health Division, Menzies School of Health Research, Charles Darwin University, Darwin, NT Australia; 5 Department of Infectious Diseases, University of Melbourne, The Doherty Institute, Melbourne, VIC Australia; 6 Department of Clinical Sciences, Liverpool School of Tropical Medicine, Liverpool, UK; 7 Department of Obstetrics and Gynaecology, Royal Darwin Hospital, Darwin, NT Australia; 8 School of Public Health, College of Medicine and Health Sciences, Wachemo University, Hosanna, Ethiopia; 9 Department of Public Health, Asrat Woldeyes Health Science Campus, Debre Berhan University, Debre Berhan, Ethiopia; 10 Discipline of Psychiatry and Mental Health, School of Clinical Medicine, Faculty of Medicine and Health, University of New South Wales, Sydney, Australia

**Keywords:** Aboriginal, Children, Hospitalisation, Postnatal mental health, Survival analysis

## Abstract

**Purpose:**

Children of mothers with postnatal mental health conditions are at increased risk of adverse health and psychosocial outcomes. This study assessed the association between postnatal mental health-related hospitalisation (MHrH) of Aboriginal and non-Aboriginal mothers and the risk of potentially avoidable hospitalisations (PAH) among their children when aged 12 to 60 months.

**Methods:**

This is a whole-population retrospective cohort study using individual-level linked records for 59,159 children born in the Northern Territory from 2002 to 2017. Children were exposed if their mothers had at least one episode of MHrH within the first postnatal year. Incidence rates of PAH were determined, and weighted multilevel mixed-effect survival analysis was used to estimate the weighted hazard ratio (wHR) of PAH associated with exposure status. Overlap weights were used for confounder adjustment.

**Results:**

The incidence rate (per 1,000 child-months) of PAH amongst 20,718 children born to Aboriginal mothers was 11.85 (95%CI: 10.27–13.67) in exposed and 8.94 (95%CI: 8.75–9.13) in non-exposed; and, amongst 38,441 children born to non-Aboriginal mothers, 1.62 (95%CI: 1.13–2.33) in exposed and 2.08 (95%CI: 2.01–2.14) in non-exposed. After adjustment, exposed children born to Aboriginal mothers had a 24% higher risk of PAH (wHR: 1.24; 95%CI: 1.04–1.47) compared to their non-exposed counterparts, with a weighted population attributable fraction (wPAF) of 0.55% (95%CI: 0.09–1.07). No corresponding difference in risk of PAH was demonstrated among children born to non-Aboriginal mothers (wHR: 0.73; 95%CI: 0.50–1.08).

**Conclusion:**

Exposed children of Aboriginal mothers experienced a higher risk of PAH compared to non-exposed children. Meaningful reductions in PAH require culturally grounded, comprehensive maternal and child health programs that respond to the broader determinants underlying preventable child hospitalisations, including maternal mental health and related issues.

**Supplementary Information:**

The online version contains supplementary material available at https://doi.org/10.1007/s00737-026-01774-3.

## Introduction

Pregnancy and childbirth carry an increased risk of mental health conditions for mothers due to unique triggers associated with pregnancy, birth, and the adjustments required after birth (Dickens and Pawluski 2018). Mental health conditions encompass mental, behavioural, and neurodevelopmental conditions characterised by clinically substantial disturbances in cognition, emotional regulation, or behaviours that reflect dysfunction in psychological, biological, and developmental processes often associated with distress and impaired functioning across personal, family, social, educational, occupational, and other important aspects of life (Organization 2022). In the postpartum period, stressors or triggers include dramatic hormonal changes and the potential for neurotransmitter imbalance; particularly serotonin and dopamine, that regulate mood (Meltzer-Brody et al. 2018, Bergunde et al. 2022). In addition, insufficient oxytocin release may reduce a mother’s stress resilience and hinder bonding with her child. These disruptions negatively affect maternal mental health (Moura et al. 2016) and indirectly contribute to maternal death (Oates 2003).

Global estimates suggest around 20% of women encounter postnatal mental health conditions (WHO 2016). Women with mental health conditions that were present before conception or developed during pregnancy are more susceptible to experiencing mental health conditions during puerperium, the period of greatest vulnerability (van der Zee-van et al. 2021). Mental health conditions during the postnatal period impose a significant economic burden: in the United Kingdom an estimated £8.1 billion has been spent annually on perinatal mental health, with a substantial proportion going toward societal costs and child-related expenses (Bauer 2015). A frequently used population-level measure of mental health is the rate of hospital admissions for these conditions. Rates for mental health-related hospitalisations (MHrH) are significantly higher in the postnatal period compared to the antenatal period (Jones et al. 2014).

Children of mothers with postnatal mental health conditions are at risk of both short and long-term consequences for their development, education, and health, including a greater risk of potentially avoidable hospitalisation (PAH) (Pierce et al. 2023, Stein et al. 2014). In Australia, a PAH is defined as an admission that could have been prevented through effective primary or community-based healthcare. PAH rates for a population are widely used as a metric for primary health care performance and the effectiveness of preventive health measures (Ansari et al. 2006, Jackson and Tobias 2001). If not managed in a timely manner, these conditions place a significant burden on children, their families, and on the health system (Demetriou et al. 2023). Research in Australia indicates that rates of PAH are high, with significant disparities between Aboriginal and non-Aboriginal populations (Butler et al. 2013, Falster et al. 2016). A study in Western Australia found a 76% increased risk of PAH in children of mothers who experienced postnatal MHrH (Pierce et al. 2023).

The Northern Territory (NT) is a sparsely populated jurisdiction stretching from the tropical north to the arid parts of central Australia. Approximately 30% of residents identify as Aboriginal and/or Torres Strait Islander (respectfully referred to hereafter as Aboriginal), which is almost 8 times higher than the national average of 3.8% (ABS 2025). About 73% of the NT’s Aboriginal population live in remote/very remote areas, compared to 15% nationally (ABS 2021). A large proportion of the NT Aboriginal population experiences socioeconomic disadvantage, partly attributed to the lasting harmful effects of Australia’s colonial history and subsequent assimilationist and racist post-colonial policies that have systematically discriminated against and marginalised Aboriginal people. Such social and historical influences underscore the potential for Aboriginal mothers and their children to be at higher risk of psychosocial and physical problems, including substance misuse (AIHW et al. 2025), poor mental health (Strengell et al. 2015), adverse child health (Menzies 2019), and to have limited access to essential services (ABS 2011 ), which requires culturally informed public health responses. Our previous study showed that Aboriginal mothers in the NT were 2.3 times more likely to experience any MHrH and 8.6 times more likely to experience substance misuse-related hospitalisation within the first postnatal year compared to their non-Aboriginal counterparts (Demeke Belay et al. 2025). Separately, the rate of PAH among Aboriginal children in the NT has been estimated to be three times higher than among non-Aboriginal children (Zhao and Zhang 2021).

While several national and state-level initiatives aim to reduce PAH rates in the general population (CEHSU 2009), it is unclear if these and other evidence-based approaches would be suited to Aboriginal children and families and the unique NT context where risk factors, including maternal mental health issues, are prevalent. The aim of this study was to evaluate the relationship between postnatal maternal MHrH and PAH in their children in the NT to inform the development of tailored, population-specific preventive health care strategies. The following research questions were developed to meet this aim: (i) Are children born to NT mothers who experienced postnatal MHrH at increased risk of PAH compared with children born to NT mothers who did not have postnatal MHrH? (ii) If so, do these risks differ between children of Aboriginal and non-Aboriginal mothers?

## Methodology

### Study design and study population

We conducted a whole population retrospective cohort study. All children born in the NT between 2002 and 2017 were included in the analysis, after exclusion of stillborn children, children who died in the first 12 months, and children born to mothers with an interstate address at birth. The results were reported in line with the Strengthening the Reporting of Observational Studies in Epidemiology (STROBE) checklist (Wardlaw et al. 2014) (see supplementary Table S1).

### Data sources and linkage

Data for this study were sourced from a repository of linked administrative data developed through the Child and Youth Development Research Partnership (CYDRP), a collaboration between Menzies School of Health Research and the NT Government. The CYDRP data repository (Extract 3) contains linkable de-identified individual-level records for all NT-residing children born from 1st January 1986 onwards, including administrative records from health, education, child protection, and justice systems. To maintain the confidentiality of study units and comply with data-use requirements, cell counts fewer than ten were suppressed and reported as “n.p.” The linkage of records for individuals was performed by SA NT DataLink using a mix of probabilistic linkage and clerical review of uncertain matches. The quality of data linkage in the NT has been estimated to have a low rate of false positive or false negative matches (0.4%) (Schneider et al. 2019). This study used linked records from three data collections: NT Perinatal Trends, NT Inpatient Activity, and NT Death Registry. Records from the NT Perinatal Trends collection come from a statutory register of information for all births in the NT including detailed information of pregnancy (Thompson et al. 2013). The NT Inpatient Activity dataset is an administrative collection that contains records on all admissions to NT public hospitals and contains details of up to 50 diagnoses coded using the International Classification of Diseases, 10th Revision, Australian Modification (ICD-10-AM) (WHO 1992). The NT Inpatient Activity dataset was used to identify inpatient episodes for children and their mothers in the study population across six public hospitals from 1st January 2000 to 31st December 2018. There is a single private hospital in the NT, which has relatively few paediatric medical admissions and no dedicated inpatient mental health beds. The NT Death Registry is a statutory collection of records of all deaths in the NT.

### Exposure variable

The exposure of interest was maternal MHrH within the first postnatal year, defined as any mental health-related conditions (ICD-10-AM diagnosis codes F00-F99) (see supplementary Table S2). Children were exposed if their mothers had at least one episode of MHrH within the first postnatal year.

### Outcome variable

Child’s potentially avoidable hospitalisation (PAH) when aged between 12 and 60 months was the outcome. We used a definition of PAH designed specifically for monitoring ambulatory care sensitive hospitalisations in a paediatric population (Anderson et al. 2012), which comprised of hospitalisation records with one or more of the following principal ICD-10-AM diagnosis codes: A00–A99, D50–D89, E00–E89, G00–G99, H00–H59, I00–I99, J00–J99, K00–K93, L00–L99, M00–M99, N00–N99, P00–P96, and R00–R99). These codes capture conditions including acute rheumatic fever, respiratory tract infections, constipation, dental problems, nutritional deficiencies, skin infections, urinary tract infections, vaccine-preventable conditions, meningitis, febrile convulsions, osteomyelitis, etc. (see supplementary Table S3). Follow-up time was calculated as months from the child’s 1st birthday to the first PAH or, for children without a PAH, treated as censored (i.e., not having an outcome) by 60 months of age. Records of children who were re-admitted to the same hospital within 48 h, or transferred to another hospital, for the same condition were considered as a single episode.

### Covariates

We selected a range of sociodemographic, psychosocial, behavioural, and perinatal covariates based on clinical relevance, expert recommendations, and existing literature (Pierce et al. 2023, Butler et al. 2013, Falster et al. 2016, Ansari et al. 2012, Harrold et al. 2014, Stamp et al. 1998, Zhou et al. 2018). Maternal Aboriginal status was obtained from records of birth in the Perinatal Trends collection and coded as Aboriginal or non-Aboriginal. Residence was defined using maternal residential location at the time of birth as recorded in the Perinatal Trends collection and categorised into the two major health service regions in the NT: the Top End region includes the Darwin Urban, Darwin Rural, Katherine, and East Arnhem districts, and the Central Australia region includes Barkly, Alice Springs Urban, and Alice Springs Rural districts (Leckning et al. 2023). Child sex (male or female) and maternal age in years at birth (< 20, 20–24, 25–29, 30–34, 35–51 or missing) were also obtained from the NT Perinatal Trends collection. Psychosocial covariates for the mother were derived from ICD-10-AM diagnosis codes for hospital admissions in the year before or during pregnancy for mental health, self-harm, and violence (see supplementary Table S4), all recorded as binary variables. Prior hospitalisations of the mother were identified based on a derived pregnancy start date (calculated by subtracting gestational age from date of birth in the perinatal records) and the date of birth. Variables for maternal risk behaviours during pregnancy, including smoking and alcohol consumption during pregnancy, were obtained from perinatal records and coded as ‘Yes’, ‘No’, or ‘Missing’. Perinatal covariates were obtained from perinatal records and included postpartum haemorrhage, renal disease, pre-existing hypertension, pre-existing diabetes, cardiac disease, urinary tract infection, intra-uterine growth retardation, antepartum haemorrhage, anaemia, fetal distress, meconium-stained liquor, obstructed labour, pre-eclampsia, gestational diabetes mellitus, and baby admission to the special care unit, all coded as ‘Yes’, ‘No’, or ‘Missing’. Additionally, the perinatal covariates included parity, gestational age (< 37 weeks, ≥ 37 weeks or missing), method of birth (normal vaginal, assisted vaginal, emergency caesarean section, or elective caesarean section), birth weight (< 2500 g, ≥ 2500 g, or missing), and antenatal care (ANC) (< 7, ≥ 7, or missing). Separate sets of covariates were used for Aboriginal and non-Aboriginal populations. For the non-Aboriginal population, covariates with low prevalence, specifically self-harm, cardiac disease, pre-existing diabetes, pre-existing hypertension, and renal disease, were excluded from the analysis (see supplementary Table S5).

### Statistical analysis

Data were cleaned, managed, and analysed using Stata 17 (Kothari 2015). Missingness in alcohol consumption and smoking during pregnancy was likely associated with social desirability bias if non-response is associated with the unobserved true value, which was handled as missing at random by including a missing indicator category to preserve statistical power. Descriptive statistics (frequencies and percentages) were computed. The incidence rate was calculated by dividing the total number of events (children’s PAH) by the total time at risk (in child-months) and reported as the number of events per 1,000 child-months (Friis and Sellers 2020). Median survival times were calculated to compare the time at which 50% of the population experienced their first PAH between the exposed and non-exposed groups (Reid 1981). Kaplan-Meier survival curves were used to compare event-free survival probabilities, defined as the proportion of individuals who remained free of PAH over time, between the exposed and non-exposed (Stalpers et al. 2018). Log-rank tests assessed whether differences in survival curves were statistically significant (Wellek 1993).

A weighted multilevel mixed-effect Weibull survival model was chosen to address the hierarchical nature of the data (Crowther 2019). We incorporated random effects to account for both individual-level (multiple events and multiple children from the same mother) and cluster-level (children having the same place of residence) correlations (Crowther 2019). We included child level random effects to control for time-invariant unobserved heterogeneity, so the hazard ratio reflects child-level associations over time (StataCorp 2013). Two models were tested to examine the risk of PAH associated with postnatal maternal MHrH: Model (I) accounting for geographic clustering based on residence, and Model (II) accounting for sibling clustering. Model fit was assessed using information criteria and the Wald chi-square test. Model II, which had the lower information criteria value and a statistically significant Wald chi-square test, was selected as the best-fitting model (Konishi and Kitagawa 2007, Lafontaine and White 1986). The risk of PAH associated with postnatal maternal MHrH was reported as a weighted hazard ratio (wHR) with 95% confidence intervals (CI), and a p-value < 0.05 was considered statistically significant. Models were developed for all children and stratified by Aboriginal status to also allow for adjustments and estimates of risk separately for Aboriginal and non-Aboriginal children.

The propensity score (PS) overlap weight was estimated through logistic regression to adjust for the confounders between the exposed and non-exposed groups (Thomas et al. 2020). We employed overlap weighting, a recently developed propensity scores analysis approach designed to mitigate the impact of extreme propensity scores (Li et al. 2019). The standardised mean difference (SMD) and variance ratio were used to check covariate balance, with values less than 0.10 and 2, respectively, considered indicative of good balance (Zhang et al. 2019). Additionally, histograms and density plots of the PS distributions were used to visually demonstrate overlap and covariate balance. The weighted population attributable fraction (wPAF) was calculated using the formula for a cohort study: wPAF = prevalence of PAH among exposed × (relative risk − 1) ÷ [1 + prevalence of exposure × (relative risk − 1)] (Miettinen 1974). In this study, the prevalence of PAH among children born to mothers with postnatal MHrH was 2.31%. The PAF estimates the proportion of PAH cases attributable to postnatal maternal MHrH that could potentially be prevented if it were eliminated by effective public health and clinical interventions. The E-value was estimated to assess how strong an unmeasured confounder would be needed, in terms of its association with both postnatal maternal MHrH and PAH, to nullify the observed association (Haneuse et al. 2019).

## Results

### Characteristics of the study cohort

A total of 59,159 children born in the NT between 2002 and 2017 were included in the analysis, after exclusion of 535 stillborn children (53% children from Aboriginal mothers), 424 children who died in the first 12 months of life (70% children born to Aboriginal mothers), and 1,342 children born to mothers with an interstate address at birth (71% children from Aboriginal mothers). Supplementary Table S5 presents the characteristics of children and their mothers based on their exposure to postnatal maternal MHrH. About 1.24% of all children were exposed to postnatal maternal MHrH, including 1.96% of 20,718 children born to Aboriginal mothers, and 0.85% of 38,441 children born to non-Aboriginal mothers. Smoking during pregnancy, alcohol consumption during pregnancy, MHrH before or during pregnancy, violence-related hospitalisation, self-harm-related hospitalisation, low birth weight, antenatal care underutilisation, and preterm birth were more frequent in the exposed compared to the unexposed groups (*p* < 0.001; see supplementary Table S5).

### Incidence of PAH

**Fig. 1:**
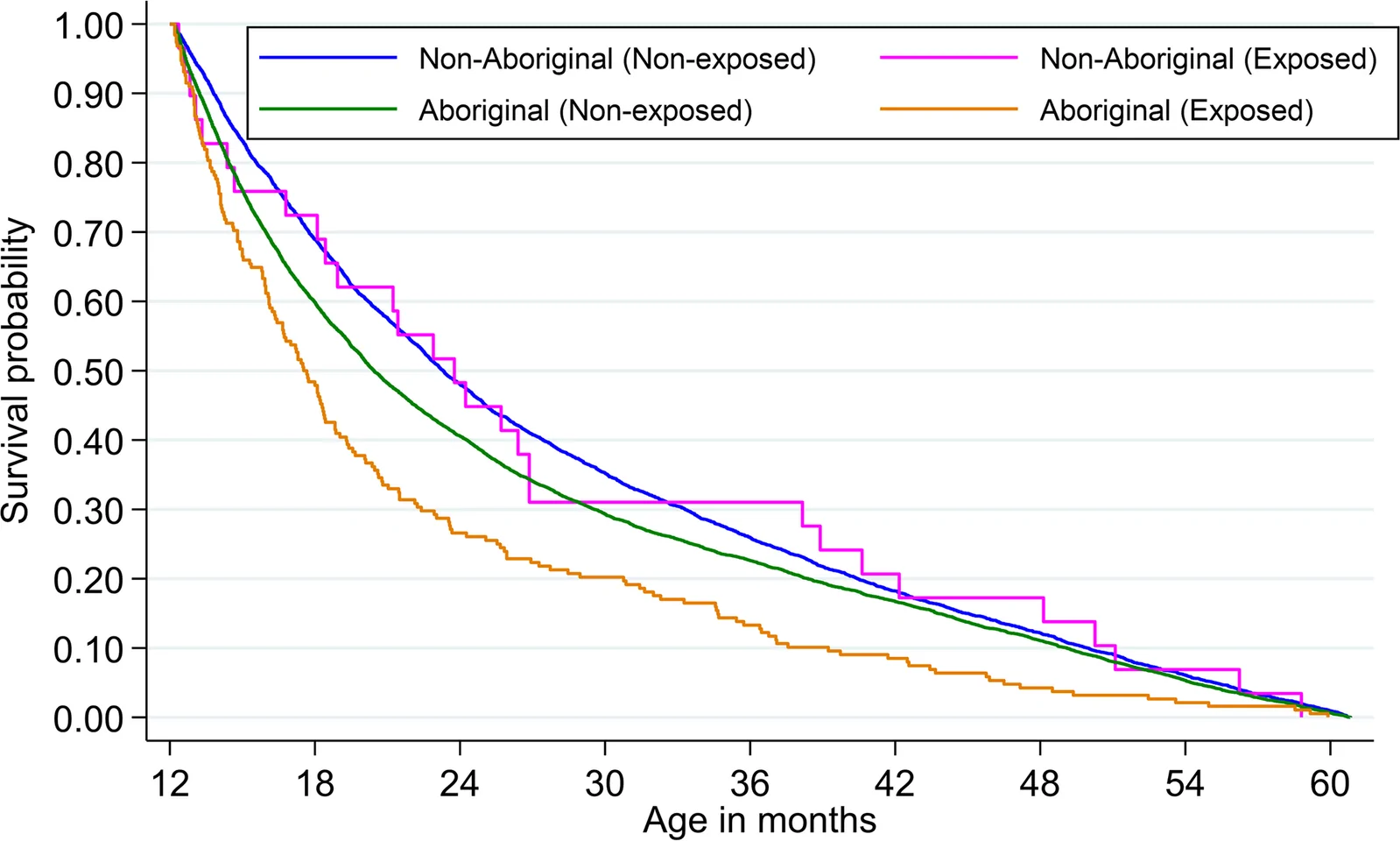
Kaplan-Meier survival probability of PAH for children born to Aboriginal and non-Aboriginal mothers in NT between 2002 and 2017, by postnatal maternal MHrH

In the overall study population, the incidence of PAH was 6.43 per 1,000 child-months (95% CI: 5.63–7.35) among exposed children and 4.12 per 1,000 child-months (95% CI: 4.05–4.19) among non-exposed children. The exposed children had a shorter median survival time to first PAH (16.8 months) than non-exposed children (20.1 months) (χ² = 43.46, degrees of freedom = 1, *p* < 0.05) (Fig. 1).

For children of Aboriginal mothers, the incidence of PAH was 11.85 per 1,000 child-months (95% CI: 10.27–13.67) when their mother had experienced postnatal MHrH and 8.94 per 1,000 child-months (95% CI: 8.57–9.13) in unexposed children. Gastroenteritis (4.49 per 1,000 child-months; 95% CI: 3.60–5.60) and skin infection (2.84 per 1,000 child-months; 95% CI: 2.16–3.73) were the most common potentially avoidable conditions (see supplementary Table S6) for details of specific potentially avoidable conditions). The exposed group had a shorter median survival time (16.4 months) than their non-exposed counterparts (19.2 months) (χ² = 30.59, degrees of freedom = 1, *p* < 0.001) (Fig. 1).

The incidence of PAH was 1.62 per 1,000 child-months (95% CI: 1.13–2.33) for children of non-Aboriginal mothers who had experienced postnatal MHrH and 2.08 per 1,000 child-months (95% CI: 2.01–2.14) in unexposed children. Gastroenteritis (0.44 per 1,000 child-months; 95% CI: 0.22–0.88) and asthma (0.22 per 1,000 child-months; 95% CI: 0.08–0.59) were the most common potentially avoidable conditions (see supplementary Table S6) for details of specific potentially avoidable conditions). The median survival time was similar between the exposed (21.4 months) and non-exposed (22.1 months) groups (χ² = 0.01, degrees of freedom = 1, *p* > 0.05) (Fig. 1).

### Risk of PAH associated with postnatal maternal MHrH

The standardised mean difference and propensity score distribution graph showed good overlap between the exposed and non-exposed groups, indicating satisfactory balance and sufficient confounding adjustment met (see supplementary Figures S1 and S2, and supplementary Tables S7 & S8).

After overlap weighting, there was no statistically significant difference in the risk of PAH between the exposed and non-exposed groups when all children were considered (wHR: 1.12; 95% CI: 0.96–1.30).

Table 1 presents the results of the multilevel mixed-effect survival analysis assessing the risk of PAH associated with postnatal maternal MHrH for children born to Aboriginal and non-Aboriginal mothers, along with the corresponding population attributable fraction (PAF) and E-values. In the stratified analysis, exposed children born to Aboriginal mothers had a 24% higher risk of PAH (wHR: 1.24; 95% CI: 1.04–1.47) compared to their non-exposed counterparts with a wPAF of 0.55% (95% CI: 0.09–1.07), indicating a half percent reduction of PAH in children if postnatal maternal MHrH had been prevented. The E-value for the observed association suggests that the confounder-child PAH association must be at least as large as 1.79 to completely explain away the observed hazard ratio. Among children born to non-Aboriginal mothers, there was no statistically significant difference in the risk of PAH between the exposed and non-exposed groups (wHR: 0.73; 95% CI: 0.50–1.08) (see Table 1).

**Table 1:** Multilevel mixed-effect survival model for children born to Aboriginal and non-Aboriginal mothers, Northern Territory, Australia, 2002–2017

Children born to Aboriginal mothers			Children born to non-Aboriginal mothers	
Exposure	cHR	wHR	cHR	wHR
MHrH				
No	1	1	1	1
Yes	1.15 (0.97–1.36)	1.24 (1.04–1.47)*	0.71 (0.49–1.03)	0.73 (0.50–1.08)
**PAF**	0.34 (-0.07-0.82)	0.55 (0.09–1.07)		
**E-value**		1.79 (1.24–2.30)		

Both models were adjusted for urinary tract infection, intra-uterine growth retardation, antepartum haemorrhage, anaemia, pre-eclampsia, residency, maternal age at birth, child sex, MHrH before and during pregnancy, violence before and during pregnancy, smoking during pregnancy, alcohol during pregnancy, antenatal care, parity, preterm birth, method of birth, birthweight, neonatal admission to special care unit, obstructed labour, fetal distress, meconium stained distress, postpartum hemorrhage and gestational diabetes. Self-harm before and during pregnancy, cardiac disease, pre-existing diabetes, pre-existing hypertension, and renal disease were additionally adjusted for the Aboriginal population. *Significant at p-value < 0.05, *wHR* weighted hazard ratio, *cHR* crude hazard ratio, *MHrH* mental health-related hospitalisation, and *PAF* population attributable fraction

## Discussion

This study estimated the risk of PAH for children born in the NT between 2002 and 2017 associated with postnatal maternal MHrH. The findings indicate that there was no statistical evidence of a difference in PAH risk between the exposed and non-exposed children in the overall population. This contrasts with a previous study conducted in Western Australia which reported an association between postnatal maternal MHrH and PAH in children (Pierce et al. 2023). This disparity may be partially explained by differences in socio-demographic characteristics and by differences in the adjustment for confounders. Additionally, the high baseline of PAHs in the NT may limit the ability to detect population-level differences between exposed and unexposed groups (Li et al. 2009). This suggests that although postnatal maternal MHrH may not be a significant independent predictor of PAH across the entire NT population, PAH remains a substantial and persistent public health issue that requires comprehensive and system-level interventions.

Stratified analysis indicates differences in risk by Aboriginal status. Among children born to non-Aboriginal mothers, the estimated association was not statistically significant before and after adjustment. The absence of association may reflect fewer structural barriers, stronger socioeconomic stability, and more access to universal maternal and child health services, which may buffer the impact of maternal mental health on engagement with preventive/primary healthcare for their children (Turney 2011). A study has shown that favourable social determinants of health, including socio-economic stability, are associated with improved access to and engagement with maternal and child health services (Austin 2023). Additionally, we have previously found that postnatal maternal MHrH was less common amongst non-Aboriginal mothers (Demeke Belay et al. 2025). The relatively low prevalence of postnatal maternal MHrH in this group may have resulted in limited power to detect an association, rather than indicating a true absence of effect, and may also partially explain the higher observed incidence of PAH among non-exposed children.

Among children born to Aboriginal mothers, the crude association between postnatal maternal MHrH and PAH was not statistically significant; however, the association became statistically significant after adjustment. Postnatal MHrH was associated with a 24% higher risk of PAH and an earlier first PAH (2.8 months) compared to their non-exposed counterparts. Our study is the first to examine and identify differences in the association between postnatal MHrH and PAH in children born to Aboriginal and non-Aboriginal mothers. This highlights the importance of accounting for population heterogeneity when examining such associations, as analyses based on aggregated data may obscure meaningful subgroup differences with important implications for targeted and equitable public health responses. The observed association between postnatal maternal MHrH and PAH in children born to Aboriginal mothers may reflect the impact of postnatal maternal mental health conditions on access to and engagement with universal maternal and child health services. Consistent with this, children born to Aboriginal mothers with postnatal MHrH experienced a significantly earlier median time to first PAH, suggesting that maternal mental health difficulties not only increase the risk of hospitalisation but also accelerate early life vulnerability. These findings highlight the need for early and timely primary maternal and child health care interventions to prevent avoidable hospitalisations. The World Health Organization and the Australian evidence recognise that maternal mental health influences access and engagement with maternal and child health services (WHO 2022, Faulks et al. 2024, Ou et al. 2010). While postnatal maternal MHrH may reduce maternal capacity to access and engage with universal maternal and child health services, weakening preventive care pathways, these mechanisms likely operate differently across populations (Slomian et al. 2019). Factors such as culture, geographic, or systemic barriers may limit service engagement for Aboriginal mothers and their children, whereas such barriers may be less pronounced in the non-Aboriginal mothers. Moreover, our previous study demonstrated that prenatal maternal mental health hospitalisation strongly predicted postnatal maternal MHrH, indicating persistence of vulnerability across the perinatal period. Thus, mothers with histories of maternal MHrH may also experience reduced engagement with maternal and child health services before and during pregnancy, contributing to adverse birth outcomes and increased risk of PAH in their children (Demeke Belay et al. 2025, Dadi et al. 2024). Consequently, the increased burden of maternal mental health problems after childbirth may reflect not only barriers to health care utilisation but also complex social and life circumstances requiring coordinated care and support from multiple health and social service providers. These challenges may be particularly relevant for children born to Aboriginal mothers, who experience a greater burden of maternal and perinatal risk factors, including alcohol use during pregnancy, that may further increase the child’s risk of PAH. Notably, substance use-related mental health conditions were the most common cause of postnatal maternal MHrH in this population. Such conditions may affect children’s health through multiple pathways, including maternal caregiving capacity, household circumstances, prenatal exposure, and lack of engagement with health and social services.

Culturally grounded, trauma-informed maternal and child health programs are critical in preventing PAH by supporting early identification and management of maternal mental health conditions and the delivery of routine child health care (Austin et al. 2022). Aboriginal-led, family-centred maternal and child health programs, such as the Australian Nurse-Family Partnership Program (Massi 2023), the Baby One Program (Campbell et al. 2018), the Building Strong Foundations program (Murphy and Best 2012), and the Derbarl Yerrigan program (Wilkes et al. 2002), address pathway linking maternal mental health challenges to PAH by strengthening access to and engagement with antenatal, postnatal, and child health care, including immunisation, health education, parenting support, early identification and referral of health concerns. However, a systematic review indicates that while existing maternal and child health programs improve engagement with services, their effectiveness in reducing preventable child hospitalisation depends on robust implementation in primary and preventive care settings (Jongen et al. 2014). Evidence from another systematic review also points to persistent gaps in service equity, quality, and continuity (Barclay et al. 2014). For example, access to maternal and child health services remains uneven, particularly in remote and rural regions where a large proportion of Aboriginal families reside, and these geographic and structural barriers further limit service utilisation (AIHW 2024). In addition, maternal mental health is not consistently embedded as an explicit clinical component of these programs (Jongen et al. 2014). This underscores the need to strengthen the accessibility, continuity, and quality of maternal and child health services by explicitly integrating maternal mental health assessment, treatment, and follow-up (Sweeney et al. 2018). Culturally grounded programs are well placed to address the underlying trauma and prevent re-traumatisation by recognising the role of culture in healing, strengthening social support and community resilience, and improving the quality and acceptability of care (Dudgeon et al. 2014, Gee et al. 2014). Evidence from First Nations health research indicates that culturally grounded, trauma-informed care can improve maternal mental and social well-being, strengthen parenting capacity, promote child health outcomes, create a safe and respectful care environment, foster trust between families and service providers, and reduce the risk of re-traumatisation (Atkinson 2002, Abuse 2013).

### Strengths and limitations

A primary strength of this study was its use of a high-quality, whole-population repository of linked longitudinal data, which permitted multilevel mixed-effects models to account for both individual and cluster-level correlations, and the overlap weights method to adjust for observed confounders. Propensity score methods are widely recommended in observational research to obtain less confounded estimates, and the reporting of the E-value is similarly encouraged to better understand the robustness of estimates (Li et al. 2019, VanderWeele and Ding 2017). However, traditional propensity score techniques often struggle with extreme propensity scores, which can introduce bias and inflate variance. To address these limitations, we employed the overlap weighting method, a recently developed approach designed to mitigate such issues (Li et al. 2019). The study also has the following limitations. First, the overall burden of PAH may be underestimated in our study design given the study population included only children who survived beyond the first year of life and PAH events occurring during the first year of life were not considered. Second, census data indicate high levels of migration amongst non-Aboriginal women of child-bearing age, who may not be included in our study (ABS 2019). However, we have focused our findings on the NT-resident population for whom there is a need to consider opportunities for prevention and intervention in the NT. Third, the use of dichotomous indicators for postnatal maternal MHrH does not capture the heterogeneity of these conditions; however, we have provided a descriptive summary of the specific conditions in supplementary Table S9 and interpreted the results accordingly. Fourth, we were unable to examine how the specific timing of postnatal maternal MHrH affected the risk for PAH in children due to the rarity of the exposure. Furthermore, mothers who experienced antenatal mental health problems may continue to experience the problem postnatally but may be less likely to be hospitalised because of geographic barriers to healthcare access, potentially leading to under-assertion of the problem postnatally and underestimation of their association with PAH. Lastly, the small E-value for the Aboriginal model suggests that the observed association may be sensitive to unmeasured confounding factors that are prevalent in the Aboriginal population, including poor housing conditions, overcrowding, and limited access to health care. These factors increase children’s exposure to infectious diseases that are typically managed in primary care, including gastroenteritis, respiratory infections, and skin infections (McDonald and Bailie 2010, Bailie et al. 2010, Carson et al. 2020). Consistent with these, gastroenteritis and skin infection were the common underlying causes of PAH observed in this study. Therefore, further research linking data on key environmental confounders is needed to better understand the observed risk level. Although postnatal maternal MHrH was statistically associated with PAH risk among children born to Aboriginal mothers, the wPAF was less than 1%. This small wPAF is consistent with PAH being shaped by interconnected social, environmental, and structural determinants rather than by a single exposure, reflecting the complex contexts in which many Aboriginal families live (Milroy 2014). Despite the broader determinants of PAH, postnatal maternal MHrH may represent a particularly important target for intervention. Maternal mental health difficulties in the postnatal period can reduce a mother’s capacity to engage effectively with universal maternal and child health services, even where such services are available. Moreover, these challenges may compound existing early life vulnerabilities, thereby accelerating the risk of PAH.

## Conclusion

All children born to mothers with postnatal MHrH experienced a higher crude incidence of and an earlier median time to PAH compared to non-exposed children; however, this is largely attributable to our findings of a higher risk of PAH in Aboriginal children. Within the Aboriginal population, the impact of postnatal maternal MHrH extends beyond immediate health consequences for the mother and may impact a child’s long-term health trajectory. Meaningful reductions in PAH for Aboriginal children require Aboriginal-led, accessible, culturally grounded, continuous, comprehensive, and quality maternal and child health programs that address the broader determinants underlying preventable child hospitalisations, including maternal mental health and related issues.

## Supplementary Information

Below is the link to the electronic supplementary material.

Supplementary Material 1

Supplementary Material 2

## Data Availability

The datasets analysed in the current study are not publicly available due to privacy, ethical, and sensitivity considerations, and access requires data custodian approval. Data requests can be made to NT Health (see https://health.nt.gov.au/data-and-research/health-data ).

## Abbreviations

CYDRP: Child and youth development research partnership
CI: Confidence interval
IC: Information criteria
ICD-10-AM: International classification of diseases, 10th revision, australian modification
MHrH: Mental health-related hospitalisation
NT: Northern territory
PAH: Potentially avoidable hospitalisations
PS: Propensity score
