# Supplementary material for: Association between maternal mental health-related hospitalisation within the first postnatal year and potentially avoidable hospitalisation among their children in the Northern Territory of Australia, using 16 years of linked administrative data
Source: Arch Womens Ment Health. 2026 Sep 30;29(5):135. doi: 10.1007/s00737-026-01774-3 (PMC13623804; doi:10.1007/s00737-026-01774-3)
Supplement: Supplementary file 2 [file 737_2026_1774_MOESM2_ESM.docx]

**Supplementary file**

Table S1: STROBE checklist used to report the findings

Table S1: ICD-10-AM codes list used to define maternal mental health-related hospitalisations, Northern Territory, Australia, 2002-2017.

Table S3: ICD-10-AM codes used to drive potentially avoidable child hospitalisations, Northern Territory, Australia, 2002-2017.

Table S4: ICD-10-AM code lists used to derive maternal violence and self-harm-related hospitalisations, Northern Territory, Australia, 2002-2017.

Table S5: Characteristics of mother-child pairs in the NT between 2002 and 2017, by MHrH.

Table S6: Incidence rate of potentially avoidable hospitalisation among children based on their exposure status, Northern Territory, Australia, 2002-2017.

Table S7: The degree of overlapping among children born to Aboriginal and non-Aboriginal mothers based on their exposure status, Northern Territory, Australia, 2002-2017.

Figure S1: The propensity score distribution using histogram and density plot among children born to Aboriginal mothers, Northern Territory, Australia, 2002-2017.

Figure S2: The propensity score distribution using histogram and density plot among children born to non-Aboriginal mothers, Northern Territory, Australia, 2002-2017.

Table S8: Standardised mean difference and variance ratio to evaluate covariate balance among children born to Aboriginal and non-Aboriginal mothers, Northern Territory, Australia, 2002-2017.

Table S9: Percentage of the specific mental health conditions among children born in the Northern Territory, Australia, 2002-2017.

**Table S1: STROBE checklist used to report the findings**

|  | | No | Recommendation | Page No |
| --- | --- | --- | --- | --- |
| **Title and abstract** | | 1 | (*a*) Indicate the study’s design with a commonly used term in the title or the abstract | 1 & 2 |
|  |  |  | (*b*) Provide in the abstract an informative and balanced summary of what was done and what was found | 2 |
| Introduction | | | | |
| Background/rationale | | 2 | Explain the scientific background and rationale for the investigation being reported | 4-5 |
| Objectives | | 3 | State-specific objectives, including any prespecified hypotheses | 5 |
| Methods | | | | |
| Study design | | 4 | Present key elements of the study design early in the paper | 6 |
| Setting | | 5 | Describe the setting, locations, and relevant dates, including periods of recruitment, exposure, follow-up, and data collection | 6-7 |
| Participants | | 6 | (*a*) Give the eligibility criteria and the sources and methods of selection of participants. Describe methods of follow-up | 7 |
|  |  |  | (*b*) For matched studies, give the matching criteria and the number of exposed and unexposed | NA |
| Variables | | 7 | Clearly define all outcomes, exposures, predictors, potential confounders, and effect modifiers. Give diagnostic criteria, if applicable | 6-8 |
| Data sources/ measurement | | 8* | For each variable of interest, give sources of data and details of methods of assessment (measurement). Describe the comparability of assessment methods if there is more than one group | 6-8 |
| Bias | | 9 | Describe any efforts to address potential sources of bias | 8-9 |
| Study size | | 10 | Explain how the study size was arrived at | 6 |
| Quantitative variables | | 11 | Explain how quantitative variables were handled in the analyses. If applicable, describe which groupings were chosen and why | 9 |
| Statistical methods | | 12 | (*a*) Describe all statistical methods, including those used to control for confounding | 8 & 9 |
|  |  |  | (*b*) Describe any methods used to examine subgroups and interactions | 9 |
|  |  |  | (*c*) Explain how missing data were addressed | 8 |
|  |  |  | (*d*) If applicable, explain how loss to follow-up was addressed | NA |
|  |  |  | (*e*) Describe any sensitivity analyses | 9 |
| Results | | | |  |
| Participants | | 13* | (a) Report numbers of individuals at each stage of study—e.g., numbers potentially eligible, examined for eligibility, confirmed eligible, included in the study, completing follow-up, and analysed | 9 |
|  |  |  | (b) Give reasons for non-participation at each stage | 9 |
|  |  |  | (c) Consider the use of a flow diagram | NA |
| Descriptive data | | 14* | (a) Give characteristics of study participants (e.g., demographic, clinical, social) and information on exposures and potential confounders | 10 |
|  |  |  | (b) Indicate the number of participants with missing data for each variable of interest | 10 |
|  |  |  | (c) Summarise follow-up time (e.g., average and total amount) | 10 |
| Outcome data | | 15* | Report numbers of outcome events or summary measures over time | 10 |
| Main results | 16 | (*a*) Give unadjusted estimates and, if applicable, confounder-adjusted estimates and their precision (eg, 95% confidence interval). Make clear which confounders were adjusted for and why they were included | | 11 & 12 |
|  |  | (*b*) Report category boundaries when continuous variables were categorized | | 10 |
|  |  | (*c*) If relevant, consider translating estimates of relative risk into absolute risk for a meaningful time period | | NA |
| Other analyses | 17 | Report other analyses done—e.g., analyses of subgroups and interactions, and sensitivity analyses | | 11 |
| Discussion | | | | |
| Key results | 18 | Summarise key results with reference to study objectives | | 12, 13 |
| Limitations | 19 | Discuss limitations of the study, considering sources of potential bias or imprecision. Discuss both the direction and the magnitude of any potential bias | | 15 |
| Interpretation | 20 | Give a cautious overall interpretation of results, considering objectives, limitations, multiplicity of analyses, results from similar studies, and other relevant evidence | | 12-14 |
| Generalisability | 21 | Discuss the generalisability (external validity) of the study results | | 12 |
| Other information | | | | |
| Funding | 22 | Give the source of funding and the role of the funders for the present study and, if applicable, for the original study on which the present article is based | | 1 |

*Give information separately for exposed and unexposed groups.

**Table S2: ICD-10-AM codes list used to define maternal mental health-related hospitalisations, Northern Territory, Australia, 2002-2017.**

| **Diagnostic categories** | | **ICD-10 code** |
| --- | --- | --- |
| **Severe** | Schizophrenia and other psychotic disorders | F20, F22, F23, F24, F28, F29 |
|  | Schizoaffective disorders | F25, F25.0, F25.1, F25.2, F25.8, F25.9 |
|  | Psychotic affective disorders | F30, F31, F32.3, F33.3 |
| Common | Non-psychotic affective disorders | F32.1, F32.2, F32.8, F32.9, F33.0, F33.0, F33.1, F33.2, F33.4, F33.8, F33.9, F34, F34.0, F34.8, F34.9, F38.0, F38.1, F38.8, F39, F53 |
|  | Neurotic and stress-related disorders | F40, F41, F42, F43, F44, F45, F48 |
| **Personality** | Personality disorder cluster A | F21, F60.0, F60.1 |
|  | Personality disorder cluster B | F60.2, F60.3, F60.30, F60.31, F60.4 |
|  | Personality disorder cluster C | F60.5, F60.6, F60.7 |
|  | Other Personality Disorders | F60, F60.8, F60.09, F60.9, F61, F62, F62.0, F62.1, F62.8, F62.9, F68.0, F68.1, F68.8, F69 |
| **Adult onset** | Other postnatal conditions | F53, F53.8, F53.9 |
|  | Other disorders not elsewhere classified | F99, F99.1 |
|  | Other Adult-onset disorders | F00, F01, F03, F04, F05, F06, F07, F09, F50, F51, F52, F54, F55, F59, F63, F64, F65, F66 |
| **Child onset** | Hyperkinetic disorders | F90 |
|  | Conduct disorders | F91, F92 |
|  | Other childhood disorders | F70, F71, F73, F78, F79, F80, F81, F82, F83, F84, F88, F89, F93, F94, F95, F98 |
| **Substance misuse-related mental health conditions** | | F10-F19, F55, F55.9 |

**Table S3: ICD-10-AM codes used to drive potentially avoidable child hospitalisations, Northern Territory, Australia, 2002-2017.**

| ***Potential avoidable hospitalisations*** | **ICD-10-AM codes** |
| --- | --- |
| Acute rheumatic fever | I00, I01, and I02 |
| Acute upper respiratory tract infection, excluding croup | J00, J01, J02, J03, J06 |
| Asthma | J45, J46 |
| Bacterial/unspecified pneumonia | J13, J14, J15, J16, J18 |
| Bronchiectasis | J47 |
| Chronic rheumatic heart disease | I05, I06, I07, I08, I09 |
| Constipation | K59.0 |
| Dental (dental caries, pulp, periodontal) | K02, K04, K05 |
| Dermatitis/eczema | L20, L21, L22, L23, L24, L25, L26, L27, L28, L29, L30 |
| Gastro-oesophageal reflux | K21 |
| Gastroenteritis | A00, A01, A02, A03, A04, A05, A06, A07, A08, A09, R11, K52.9 |
| Nutritional deficiency | E40, E41, E42, E43, E44, E45, E46, E50, E51, E52, E53, E54, E55, E56, E58, E59, E60, E61, E62, E63, E64, D50, D51, D52, D53 |
| Otitis media | H65, H66, H67 |
| Skin infection | L00, L01, L02, L03, L04, L05, L08, L98.0, J34.0, H01.0, H00.0 |
| Urinary tract infection (≥5 years of age) | N10, N12, N13.6, N30.0, N30.9, N39.0 |
| Vaccine-preventable diseases | A33, A34, A35, A36, A37, A80, B05, B06, B16, B18.0, B18.1, B26, M01.4, P35.0 |
| Acute bronchiolitis | J21 |
| Bacterial meningitis | G00, G01 |
| Croup, acute laryngitis, tracheitis | J04, J05.0 |
| Febrile convulsions | R56.0 |
| Meningococcal disease | A39 |
| Osteomyelitis | M86 |
| Tuberculosis | A15, A16, A17, A18, A19 |
| Viral infection of an unspecified site | B34 |
| Viral pneumonia | J12, J10.0, J11.0 |
| Viral/other/unspecified meningitis | A87, G02, G03 |

**Table S4: ICD-10-AM code lists used to derive maternal violence and self-harm-related hospitalisations, Northern Territory, Australia, 2002-2017.**

| **Violence** | **Types of adversity-related hospitalisations** | **ICD-10-AM codes** |
| --- | --- | --- |
|  | **Violence other than assault** | |
|  | Maltreatment syndromes | T74.0,1,2,3,8,9 |
|  | Effects of other deprivation (extreme neglect) | T73.0, |
|  | Events of undetermined intent | Y20-Y34 |
|  | Examination and observation following other inflicted injuries | Z04.5 |
|  | Examination and observation for other reasons: request for expert evidence | Z04.8 |
|  | Examination and observation for other reasons | Z04.0-Z04.9 |
|  | Perpetrator of neglect and other maltreatment syndromes | Y06, Y07 |
|  | **Assault** | |
|  | Assault by bodily force and sexual assault | Y04, Y05 |
|  | Other types of assault | X85-Y03, Y08-Y09 |
|  | Personal history of violence | Z91.5 |
| **Self-harm** | Self-harm | |
|  | Sequelae of intentional self-harm | Y87 |
|  | Intentional self-harm | X6, X7, X80-X84 |
|  | Personal history of self-harm | Z91.5 |

**Table S5: Characteristics of mother-child pairs in the NT between 2002 and 2017, by MHrH.**

| **Covariates** | **Children born to Aboriginal mothers (N = 20,718)** | | | **Children born to non-Aboriginal mothers (N = 38,441)** | | | **All children born in the NT (N = 59,159)** | | |
| --- | --- | --- | --- | --- | --- | --- | --- | --- | --- |
|  | Non-exposed | Exposed | p-value | Non-exposed | Exposed | p-value | Non-exposed | Exposed | p-value |
| ***Socio-demographic characteristics*** | | | | | |  |  |  |  |
| **Residency** |  |  | **<0.001** |  |  | **>0.05** |  |  | **<0.001** |
| Top End Australia | 14,089 (69.36) | 198 (48.77) |  | 32,353 (84.88) | 272 (83.69) |  | 46,442 (79.49) | 470 (64.30) |  |
| Central Australia | 6,223 (30.64) | 208 (51.23) |  | 5,763 (15.12) | 53 (16.31) |  | 11,986 (20.51) | 261 (35.70) |  |
| **Maternal age at birth** |  |  | **<0.001** |  |  | **>0.05** |  |  | **<0.05** |
| <20 | 4,646 (22.87) | 56 (13.79) |  | 1,275 (3.35) | 17 (5.23) |  | 5,921 (10.13) | 73 (9.99) |  |
| 20-24 | 6,515 (32.07) | 129 (31.77) |  | 5,692 (14.93) | 60 (18.46) |  | 12,207 (20.89) | 189 (25.85) |  |
| 25-29 | 4,763 (23.45) | 99 (24.38) |  | 11,049 (28.99) | 96 (29.54) |  | 15,812 (27.06) | 195 (26.68) |  |
| 30-34 | 2,890 (14.23) | 78 (19.21) |  | 12,338 (32.37) | 91 (28.00) |  | 15,228 (26.06) | 169 (23.12) |  |
| 35-51 | 1,498 (7.37) | 44 (10.84) |  | 7,761 (20.36) | 61 (18.77) |  | 9,259 (15.85) | 105 (14.36) |  |
| Missing | 0 (0.00) | 0 (0.00) |  | n.p. | n.p. |  | 0 (0.00) | 0 (0.00) |  |
| **Child sex** |  |  | **>0.05** |  |  | **>0.05** |  |  | **>0.05** |
| Male | 10,474 (51.57) | 218 (53.69) |  | 19,518 (51.21) | 160 (49.23) |  | 29,992 (51.33) | 378 (51.71) |  |
| Female | 9,833 (48.41) | 188 (46.31) |  | 18,587 (48.76) | 165 (50.77) |  | 28,420 (48.64) | 353 (48.29) |  |
| Missing | n.p. | n.p. |  | 11(0.03) | n.p. |  | 16 (0.03) | 0 (0.00) |  |
| ***History of maternal admissions a year before or during pregnancy*** | | | | | |  |  |  |  |
| **MHrH** |  |  | **<0.001** |  |  | **<0.001** |  |  | **<0.001** |
| No | 19,657 (96.78) | 276 (67.98) |  | 37,742 (99.02) | 273 (84.00) |  | 57,399 (98.24) | 549 (75.10) |  |
| Yes | 655 (3.22) | 130 (32.02) |  | 374 (0.98) | 52 (16.00) |  | 1,029 (1.76) | 182 (24.90) |  |
| **Violence** |  |  | **<0.001** |  |  | **<0.001** |  |  | **<0.001** |
| No | 19,910 (98.02) | 351 (86.45) |  | 37,954 (99.57) | 312 (96.00) |  |  |  |  |
| Yes | 402 (1.98) | 55 (13.55) |  | 162 (0.43) | 13 (4.00) |  |  |  |  |
| **Self-harm** |  |  | **<0.001** |  |  | **<0.001** |  |  | **<0.001** |
| No | 20,230 (99.60) | 391 (96.31) |  | n.p. | n.p. |  | 58,310 (99.80) | 710 (97.13) |  |
| Yes | 82 (0.40) | 15 (3.69) |  | n.p. | n.p. |  | 118 (0.20) | 21 (2.87) |  |
| ***Behavioural characteristics of the mother*** | | | | | |  |  |  |  |
| **Smoking during pregnancy** |  |  | **<0.001** |  |  | **<0.001** |  |  | **<0.001** |
| No | 8,098 (39.87) | 129 (31.77) |  | 28,324 (74.31) | 199 (61.23) |  | 36,422 (62.34) | 328 (44.87) |  |
| Yes | 9,014 (44.38) | 221 (54.43) |  | 4,997 (13.11) | 93 (28.62) |  | 14,011 (23.98) | 314 (42.95) |  |
| Missing | 3,200 (15.75) | 56 (13.79) |  | 4,795 (12.58) | 33 (10.15) |  | 7,995 (13.68) | 89 (12.18) |  |
| **Alcohol during pregnancy** |  |  | **<0.001** |  |  | **<0.05** |  |  | **<0.001** |
| No | 13,677 (67.33) | 194 (47.78) |  | 30,617 (80.33) | 236 (72.62) |  | 44,294 (75.81) | 430 (58.82) |  |
| Yes | 1,986 (9.78) | 122 (30.05) |  | 1,691 (4.44) | 23 (7.08) |  | 3,677 (6.29) | 145 (19.84) |  |
| Missing | 4,649 (22.89) | 90 (22.17) |  | 5,808 (15.24) | 66 (20.31) |  | 10,457 (17.90) | 156 (21.34) |  |
| ***Perinatal characteristics*** | | | | | |  |  |  |  |
| **Antenatal care** |  |  | **<0.001** |  |  | **<0.001** |  |  | **<0.001** |
| < 7 | 6,859 (33.77) | 171 (42.12) |  | 5,020 (13.17) | 70 (21.54) |  | 11,879 (20.33) | 241 (32.97) |  |
| >=7 | 13,001 (64.01) | 222 (54.68) |  | 32,876 (86.25) | 251 (77.23) |  | 45,877 (78.52) | 473 (64.71) |  |
| Missing | 452 (2.23) | 13 (3.20) |  | n.p. | n.p. |  | 672 (1.15) | 17 (2.33) |  |
| **Parity (median (IQR))** | 1 (0-2) | 1 (0-3) |  | 1 (0-1) | 0 (0-2) |  | 1 (0-2) | 1 (0-2) |  |
| **Gestational age** |  |  | **<0.001** |  |  | **<0.001** |  |  | **<0.001** |
| <37 weeks | 2,906 (14.31) | 86 (21.18) |  | 2,574 (6.75) | 46 (14.15) |  | 5,480 (9.38) | 132 (18.06) |  |
| ≥37 weeks | 17,403 (85.68) | 320 (78.82) |  | 35,542 (93.25) | 279 (85.85) |  | 52,945 (90.62) | 599 (81.94) |  |
| **Method of birth** |  |  | **<0.05** |  |  | **<0.001** |  |  | **<0.001** |
| Normal vaginal | 13,442 (66.18) | 244 (60.10) |  | 22,214 (58.28) | 171 (52.62) |  | 35,656 (61.03) | 415 (56.77) |  |
| Assisted vaginal | 1,160 (5.71) | 22 (5.42) |  | 3,769 (9.89) | 29 (8.92) |  | 4,929 (8.44) | 51 (6.98) |  |
| Elective Caesarean section | 2,045 (10.07) | 49 (12.07) |  | 6,341 (16.64) | 48 (14.77) |  | 8,386 (14.35) | 97 (13.27) |  |
| Emergency Caesarean section | 3,665 (18.04) | 91 (22.41) |  | 5,792 (15.20) | 77 (23.69) |  | 9,457 (16.19) | 168 (22.98) |  |
| **Birth weight** |  |  | **<0.001** |  |  | **<0.001** |  |  | **<0.001** |
| <2500 grams | 2,674 (13.16) | 99 (24.38) |  | 2,095 (5.50) | 40 (12.31) |  | 4,769 (8.16) | 139 (19.02) |  |
| ≥2500 grams | 17,638 (86.84) | 307 (75.62) |  | 36,017 (94.49) | 285 (87.69) |  | 53,655 (91.83) | 592 (80.98) |  |
| Missing | 0 (0.00) | 0 (0.00) |  | n.p. | n.p. |  | 54,561 (93.38) | 681 (93.16) |  |
| **SCN admission** |  |  | **<0.001** |  |  | **<0.001** |  |  | **<0.001** |
| No | 15,584 (76.72) | 261 (64.29) |  | 32,167 (84.39) | 242 (74.46) |  | 47,751 (81.73) | 503 (68.81) |  |
| Yes | 4,608 (22.69) | 143 (35.22) |  | 5,510 (14.46) | 82 (25.23) |  | 10,118 (17.32) | 225 (30.78) |  |
| Missing | n.p. | n.p. |  | n.p. | n.p. |  | n.p. | n.p. |  |
| **Obstructed labour** |  |  | **>0.05** |  |  | **>0.05** |  |  | **>0.05** |
| No | 19,187 (94.46) | 379 (93.35) |  | 35,493 (93.12) | 298 (91.69) |  | 54,561 (93.38) | 681 (93.16) |  |
| Yes | 1,125 (5.54) | 27 (6.65) |  | 2,623 (6.88) | 27 (8.31) |  | 3,867 (6.62) | 50 (6.84) |  |
| **Fetal distress** |  |  | **<0.05** |  |  | **<0.001** |  |  | **<0.001** |
| No | 17,792 (87.59) | 339 (83.50) |  | 33,949 (89.07) | 262 (80.62) |  | 51,741 (88.56) | 601 (82.22) |  |
| Yes | 2,520 (12.41) | 67 (16.50) |  | 4,167 (10.93) | 63 (19.38) |  | 6,687 (11.44) | 130 (17.78) |  |
| **Meconium-stained liquor** |  |  | **<0.05** |  |  | **>0.05** |  |  | **<0.001** |
| No | 17,585 (86.57) | 333 (82.02) |  | 34,096 (89.45) | 280 (86.15) |  | 51,681 (88.45) | 613 (83.86) |  |
| Yes | 2,727 (13.43) | 73 (17.98) |  | 4,020 (10.55) | 45 (13.85) |  | 6,747 (11.55) | 118 (16.14) |  |
| **Postpartum haemorrhage** |  |  | **>0.05** |  |  | **<0.05** |  |  | **<0.05** |
| No | 16,764 (82.53) | 337 (83.00) |  | 33,513 (87.92) | 268 (82.46) |  | 50,277 (86.05) | 605 (82.76) |  |
| Yes | 3,548 (17.47) | 69 (17.00) |  | 4,603 (12.08) | 57 (17.54) |  | 8,151 (13.95) | 126 (17.24) |  |
| **GDM** |  |  | **<0.05** |  |  | **>0.05** |  |  | **<0.05** |
| No | 18,280 (90.00) | 352 (86.70) |  | 35,203 (92.36) | 295 (90.77) |  | 53,483 (91.54) | 647 (88.51) |  |
| Yes | 2,032 (10.00) | 54 (13.30) |  | 2,913 (7.64) | 30 (9.23) |  | 4,945 (8.46) | 84 (11.49) |  |
| **Pre-eclampsia** |  |  | **>0.05** |  |  | **<0.05** |  |  | **<0.05** |
| No | 19,884 (97.89) | 393 (96.80) |  | 37,555 (98.53) | 315 (96.92) |  | 57,439 (98.31) | 708 (96.85) |  |
| Yes | 428 (2.11) | 13 (3.20) |  | 561 (1.47) | 10 (3.08) |  | 989 (1.69) | 23 (3.15) |  |
| **Maternal anaemia** |  |  | **>0.05** |  |  | **>0.05** |  |  | **<0.001** |
| No | 18,293 (90.06) | 357 (87.93) |  | 37,269 (97.78) | 313 (96.31) |  | 55,562 (95.09) | 670 (91.66) |  |
| Yes | 2,019 (9.94) | 49 (12.07) |  | 847 (2.22) | 12 (3.69) |  | 2,866 (4.91) | 61 (8.34) |  |
| **Antepartum haemorrhage** |  |  | **>0.05** |  |  | **<0.05** |  |  | **<0.05** |
| No | 20,038 (98.65) | 396 (97.54) |  | 37,442 (98.23) | 313 (96.31) |  | 57,480 (98.38) | 709 (96.99) |  |
| Yes | 274 (1.35) | 10 (2.46) |  | 674 (1.77) | 12 (3.69) |  | 948 (1.62) | 22 (3.01) |  |
| **IUGR** |  |  | **<0.001** |  |  | **<0.05** |  |  | **<0.001** |
| No | 19,323 (95.13) | 365 (89.90) |  | 37,423 (98.18) | 314 (96.62) |  | 56,746 (97.12) | 679 (92.89) |  |
| Yes | 989 (4.87) | 41 (10.10) |  | 693 (1.82) | 11 (3.38) |  | 1,682 (2.88) | 52 (7.11) |  |
| **Maternal UTI** |  |  | **>0.05** |  |  | **>0.05** |  |  | **<0.001** |
| No | 19,089 (93.98) | 374 (92.12) |  | n.p. | n.p. |  | 56,882 (97.35) | 696 (95.21) |  |
| Yes | 1,223 (6.02) | 32 (7.88) |  | n.p. | n.p. |  | 1,546 (2.65) | 35 (4.79) |  |
| **Maternal cardiac disease** |  |  | **>0.05** |  |  | **>0.05** |  |  | **<0.05** |
| No | 19,187 (94.46) | 379 (93.35) |  | n.p. | n.p. |  | 57,059 (97.66) | 702 (96.03) |  |
| Yes | 1,125 (5.54) | 27 (6.65) |  | n.p. | n.p. |  | 1,369 (2.34) | 29 (3.97) |  |
| **Pre-existing diabetes** |  |  | **>0.05** |  |  | **<0.05** |  |  | **<0.001** |
| No | 19,913 (98.04) | 394 (97.04) |  | n.p. | n.p. |  | 57,911 (99.12) | 715 (97.81) |  |
| Yes | 399 (1.96) | 12 (2.96) |  | n.p. | n.p. |  | 517 (0.88) | 16 (2.19) |  |
| **Pre-existing hypertension** |  |  | **<0.001** |  |  | **>0.05** |  |  | **<0.001** |
| No | 20,023 (98.58) | 390 (96.06) |  | n.p. | n.p. |  | 57,919 (99.13) | 713 (97.54) |  |
| Yes | 289 (1.42) | 16 (3.94) |  | n.p. | n.p. |  | 509 (0.87) | 18 (2.46) |  |
| **Maternal renal disease** |  |  | **>0.05** |  |  | **<0.001** |  |  | **<0.05** |
| No | 19,833 (97.64) | 394 (97.04) |  | n.p. | n.p. |  | 57,839 (98.99) | 716 (97.95) |  |
| Yes | 479 (2.36) | 12 (2.96) |  | n.p. | n.p. |  | 589 (1.01) | 15 (2.05) |  |

**n.p. = results not presented due to low cell count values; MHrH: Mental health-related hospitalisation; IQR: Inter quantile range; SCN: Special care nursery; IUGR: Intrauterine growth retardation; GDM: Gestational diabetes mellitus; UTI: Urinary tract infection**

Table S6: Incidence rate of potentially avoidable hospitalisation among children based on their exposure status, Northern Territory, Australia, 2002-2017. Suppress

| **Conditions** | **MHrH** | **Children born to Aboriginal mothers** | | | **Children born to non-Aboriginal mothers** | | |
| --- | --- | --- | --- | --- | --- | --- | --- |
|  |  | Child-months | PAH (yes) | IR (95% CI)/10^3^ child months | Child-months | PAH (yes) | IR (95% CI)/10^3^ child months |
| Acute rheumatic fever | No | 974,910.83 | 62 | 0.06 (0.05 – 0.08) | 2,111,502.40 | 0 | 0.00 |
|  | Yes | 18,529.69 | n.p. | 0.16 (0.05 – 0.50) | 18,059.97 | 0 | 0.00 |
|  | Total | 993,440.53 | 65 | 0.07 (0.05 – 0.08) | 2,129,562.40 | 0 | 0.00 |
| Acute upper respiratory tract infection, excluding croup | No | 965,017.37 | 839 | 0.87 (0.81 – 0.93) | 2,107,033.30 | 645 | 0.31 (0.28 – 0.33) |
|  | Yes | 18,069.64 | 35 | 1.94 (1.39 – 2.70) | 18,026.03 | n.p. | 0.17 (0.05 – 0.52) |
|  | Total | 983,087.01 | 874 | 0.89 (0.83 – 0.95) | 2,125,059.30 | 648 | 0.30 (0.28 – 0.33) |
| Asthma | No | 971,605.71 | 344 | 0.35 (0.32 – 0.39) | 2,107,938.00 | 524 | 0.25 (0.23 – 0.27) |
|  | Yes | 18,416.03 | 11 | 0.60 (0.33 – 1.08) | 18,026.10 | n.p. | 0.22 (0.08 – 0.59) |
|  | Total | 990,021.74 | 355 | 0.36 (0.32 – 0.40) | 2,125,964.10 | 528 | 0.25 (0.23 – 0.27) |
| Bacterial/unspecified pneumonia | No | 956,304.80 | 1,518 | 1.59 (1.51 – 1.67) | 2,108,944.40 | 467 | 0.22 (0.20 – 0.24) |
|  | Yes | 17,906.04 | 39 | 2.18 (1.59 – 2.98) | 18,050.17 | n.p. | 0.17 (0.05 – 0.52) |
|  | Total | 974,210.84 | 1,557 | 1.60 (1.52 – 1.68) | 2,126,994.60 | 470 | 0.22 (0.20 – 0.24) |
| Bronchiectasis | No | 972,082.63 | 288 | 0.30 (0.26 – 0.33) | 2,111,263.10 | 30 | 0.01 (0.01 – 0.02) |
|  | Yes | 18,418.73 | 15 | 0.81 (0.49 – 1.35) | 18,059.97 | 0 | 0.00 |
|  | Total | 990,501.36 | 303 | 0.31 (0.27 – 0.34) | 2,129,323.10 | 30 | 0.01 (0.01 – 0.02) |
| Chronic rheumatic heart disease | No | 975,026.85 | n.p. | 0.01 (0.00 – 0.02) | 2,111,502.40 | 0 | 0.00 |
|  | Yes | 18,536.06 | 0 | 0.00 | 18,059.97 | 0 | 0.00 |
|  | Total | 993,562.91 | n.p. | 0.01 (0.00 – 0.02) | 2,129,562.40 | 0 | 0.00 |
| Constipation | No | 974,745.97 | 26 | 0.03 (0.02 – 0.04) | 2,111,139.60 | 52 | 0.02 (0.02 – 0.03) |
|  | Yes | 18,536.06 | 0 | 0.00 | 18,059.97 | 0 | 0.00 |
|  | Total | 993,282.03 | 26 | 0.03 (0.02 – 0.04) | 2,129,199.60 | 52 | 0.02 (0.02 – 0.03) |
| Dental problems | No | 972,660.71 | 1,058 | 1.09 (1.02 – 1.16) | 2,110,851.20 | 385 | 0.18 (0.17 – 0.20) |
|  | Yes | 18,474.19 | 15 | 0.81 (0.49 – 1.35) | 18,034.97 | n.p. | 0.11 (0.03 – 0.44) |
|  | Total | 991,134.90 | 1,073 | 1.08 (1.02 – 1.15) | 2,128,886.10 | 387 | 0.18 (0.16 – 0.20) |
| Dermatitis/eczema | No | 974,746.55 | 20 | 0.02 (0.01 – 0.03) | 2,111,217.00 | 27 | 0.01 (0.01 – 0.02) |
|  | Yes | 18,478.33 | n.p. | 0.11 (0.03 – 0.43) | 18,050.40 | n.p. | 0.06 (0.01 – 0.39) |
|  | Total | 993,224.88 | 22 | 0.02 (0.01 – 0.03) | 2,129,267.40 | 28 | 0.01 (0.01 – 0.02) |
| Gastro-oesophageal reflux | No | 975,032.15 | n.p. | 0.00 (0.00 – 0.01) | 2,111,454.20 | n.p. | 0.00 (0.00 – 0.01) |
|  | Yes | 18,536.06 | 0 | 0.00 | 18,059.97 | 0 | 0.00 |
|  | Total | 993,568.21 | n.p. | 0.00 (0.00 – 0.01) | 2,129,514.20 | n.p. | 0.00 (0.00 – 0.01) |
| Nutritional deficiency | No | 971,150.18 | 331 | 0.34 (0.31 – 0.38) | 2,111,339.50 | 29 | 0.01 (0.01 – 0.02) |
|  | Yes | 18,456.07 | n.p. | 0.33 (0.15 – 0.72) | 18,059.97 | 0 | 0.00 |
|  | Total | 989,606.25 | 337 | 0.34 (0.31 – 0.38) | 2,129,399.50 | 29 | 0.01 (0.01 – 0.02) |
| Otitis media | No | 971,428.41 | 462 | 0.48 (0.43 – 0.52) | 2,108,997.2 | 397 | 0.19 (0.17 – 0.21) |
|  | Yes | 18,211.66 | 21 | 1.15 (0.75 – 1.77) | 18,059.97 | n.p. | 0.17 (0.05 – 0.52) |
|  | Total | 989,640.07 | 483 | 0.49 (0.45 – 0.53) | 2,127,057.2 | 400 | 0.19 (0.17 – 0.21) |
| Skin infection | No | 958,238.74 | 2,306 | 2.41 (2.31 – 2.51) | 2,110,328.9 | 387 | 0.183 (0.166 – 0.203) |
|  | Yes | 17,970.46 | 51 | 2.84 (2.16 – 3.73) | 18,059.97 | n.p. | 0.166 (0.054 – 0.515) |
|  | Total | 976,209.20 | 2,357 | 2.41 (2.32 – 2.51) | 2,128,388.9 | 390 | 0.183 (0.166 – 0.202) |
| Urinary tract infection | No | 972,282.86 | 222 | 0.23 (0.20 – 0.26) | 2,110,805.8 | 130 | 0.0616 (0.0519 – 0.0731) |
|  | Yes | 18,454.93 | n.p. | 0.38 (0.18 – 0.80) | 18,059.97 | n.p. | 0.1107 (0.0277 – 0.4428) |
|  | Total | 990,737.78 | 229 | 0.23 (0.20 – 0.26) | 2,128,865.7 | 132 | 0.0620 (0.0523 – 0.0735) |
| Vaccine-preventable diseases | No | 975,028.12 | n.p. | 0.01 (0.00 – 0.01) | 2,111,456.7 | n.p. | 0.00332 (0.00158 – 0.00695) |
|  | Yes | 18,536.06 | 0 | 0.00 | 18,059.97 | 0 | 0.00 |
|  | Total | 993,564.18 | n.p. | 0.01 (0.00 – 0.01) | 2,129,516.7 | n.p. | 0.00329 (0.00157 – 0.00690) |
| Gastroenteritis | No | 943,296.69 | 2,642 | 2.80 (2.70 – 2.91) | 2,107,332.8 | 837 | 0.3972 (0.3712 – 0.4250) |
|  | Yes | 17,376.74 | 78 | 4.49 (3.60 – 5.60) | 17,980.3 | n.p. | 0.4449 (0.2225 – 0.8897) |
|  | Total | 960,673.43 | 2,720 | 2.83 (2.73 – 2.94) | 2,125,313.2 | 845 | 0.3976 (0.3717 – 0.4253) |
| Acute bronchiolitis | No | 963,329.13 | 753 | 0.78 (0.73 – 0.84) | 2,108,830.2 | 314 | 0.1489 (0.1333 – 0.1663) |
|  | Yes | 17,911.57 | 29 | 1.62 (1.13 – 2.33) | 18,013.7 | n.p. | 0.1665 (0.0537 – 0.5164) |
|  | Total | 981,240.71 | 782 | 0.80 (0.74 – 0.85) | 2,126,844 | 317 | 0.1490 (0.1335 – 0.1664) |
| Bacterial meningitis | No | 974,955.39 | 13 | 0.01 (0.01 – 0.02) | 2,111,500.3 | n.p. | 0.00284 (0.00128 – 0.00633) |
|  | Yes | 18,536.06 | 0 | 0.00 | 18,059.965 | 0 | 0.00 |
|  | Total | 993,491.45 | 13 | 0.01 (0.01 – 0.02) | 2,129,560.3 | n.p. | 0.00282 (0.00127 – 0.00627) |
| Febrile convulsions | No | 973,517.12 | 133 | 0.14 (0.12 – 0.16) | 2,110,596.5 | 145 | 0.0687 (0.0584 – 0.0808) |
|  | Yes | 18,536.06 | n.p. | 0.11 (0.03 – 0.43) | 18,059.965 | n.p. | 0.0554 (0.00780 – 0.3931) |
|  | Total | 992,053.18 | 135 | 0.14 (0.11 – 0.16) | 2,128,656.5 | 146 | 0.0686 (0.0583 – 0.0807) |
| Meningococcal disease | No | 974,888.65 | 15 | 0.02 (0.01 – 0.03) | 2,111,499.3 | n.p. | 0.000947 (0.000237 – 0.00379) |
|  | Yes | 18,536.06 | 0 | 0.00 | 18,059.965 | 0 | 0.00 |
|  | Total | 993,424.71 | 15 | 0.02 (0.01 – 0.03) | 2,129,559.3 | n.p. | 0.000939 (0.000235 – 0.00376) |
| Osteomyelitis | No | 974,827.96 | 45 | 0.05 (0.03 – 0.06) | 2,111,478.1 | 11 | 0.00521 (0.00289 – 0.00941) |
|  | Yes | 18,505.26 | n.p. | 0.05 (0.01 – 0.38) | 18,059.965 | n.p. | 0.05537 (0.00780 – 0.39308) |
|  | Total | 993,333.22 | 46 | 0.05 (0.03 – 0.06) | 2,129,538.1 | 12 | 0.00564 (0.00320 – 0.00992) |
| Tuberculosis | No | 974,924.75 | n.p. | 0.01 (0.00 – 0.02) | 2,111,502.4 | n.p. | 0.000947 (0.000237 – 0.003787) |
|  | Yes | 18,536.06 | 0 | 0.00 | 18,059.965 | 0 | 0.00 |
|  | Total | 993,460.81 | n.p. | 0.01 (0.00 – 0.02) | 2,129,562.4 | n.p. | 0.000939 (0.000235 – 0.003755) |
| Viral infection of an unspecified site | No | 972,220.69 | 312 | 0.32 (0.29 – 0.36) | 2,109,737.6 | 354 | 0.1678 (0.1512 – 0.1862) |
|  | Yes | 18,499.46 | n.p. | 0.32 (0.15 – 0.72) | 18,059.965 | 0 | 0.00 |
|  | Total | 990,720.15 | 318 | 0.32 (0.29 – 0.36) | 2,127,797.5 | 354 | 0.1664 (0.1499 – 0.1846) |
| Viral pneumonia | No | 971,985.14 | 241 | 0.25 (0.22 – 0.28) | 2,110,646.5 | 158 | 0.07486 (0.06405 – 0.08749) |
|  | Yes | 18,459.97 | n.p. | 0.49 (0.25 – 0.94) | 18,057.865 | n.p. | 0.05538 (0.00780 – 0.39313) |
|  | Total | 990,445.11 | 250 | 0.25 (0.22 – 0.29) | 2,128,704.4 | 159 | 0.07469 (0.06394 – 0.08725) |
| Viral/other/unspecified meningitis | No | 974,707.85 | 29 | 0.03 (0.02 – 0.04) | 2,111,482.6 | 10 | 0.004736 (0.002548 – 0.008802) |
|  | Yes | 18,530.17 | n.p. | 0.11 (0.03 – 0.43) | 18,059.965 | 0 | 0.00 |
|  | Total | 993,238.02 | 31 | 0.03 (0.02 – 0.04) | 2,129,542.5 | 10 | 0.004696 (0.002527 – 0.008727) |
| Croup, acute laryngitis, tracheitis | No | 973,804.15 | 135 | 0.14 (0.12 – 0.16) | 2,110,008.6 | 293 | 0.138862 (0.123839 – 0.155708) |
|  | Yes | 18,536.06 | n.p. | 0.05 (0.01 – 0.38) | 18,059.532 | n.p. | 0.166117 (0.053576 – 0.515058) |
|  | Total | 992,340.21 | 136 | 0.14 (0.12 – 0.16) | 2,128,068.1 | 296 | 0.139093 (0.124117 – 0.155877) |
| Injury | No | 970,292.29 | 780 | 0.80 (0.75 – 0.86) | 2,109,706.6 | 568 | 0.269232 (0.247977 – 0.292309) |
|  | Yes | 18,428.00 | 20 | 1.09 (0.70 – 1.68) | 18,027.365 | n.p. | 0.221885 (0.083277 – 0.591192) |
|  | Total | 988,720.29 | 800 | 0.81 (0.75 – 0.87) | 2,127,733.9 | 572 | 0.268831 (0.247678 – 0.291789) |
| Potentially avoidable hospitalisations | No | 894,564.74 | 7,949 | 8.89 (8.69 – 9.08) | 2,088,651 | 4,343 | 2.0793 (2.0184 – 2.1421) |
|  | Yes | 15,868.45 | 188 | 11.85 (10.27 – 13.67) | 17,862.232 | 29 | 1.6235 (1.1282 – 2.3363) |
|  | Total | 910,433.19 | 8,137 | 8.94 (8.75 – 9.13) | 2,106,513.3 | 4,372 | 2.0755 (2.0148 – 2.1379) |

Table S7: The degree of overlapping among children born to Aboriginal and non-Aboriginal mothers based on their exposure status, Northern Territory, Australia, 2002-2017.

| **MHrH** | **Children born to Aboriginal mothers** | | | **Children born to non-Aboriginal mothers** | | |
| --- | --- | --- | --- | --- | --- | --- |
|  | Off support | On support | Total | Off support | On support | Total |
| No | 945 (2.84) | 32,338 (97.16) | 33,283 (100.00) | 740 (1.73) | 41,934 (98.27) | 42,674 (100.00) |
| Yes | 27 (2.90) | 903 (97.10) | 930 (100.00) | 5 (1.36) | 362 (98.64) | 367 (100.00) |
| Total | 972 (2.84) | 33,241 (97.16) | 34,213 (100.00) | 745 (1.73) | 42,296 (98.27) | 43,041 (100.00) |

Figure S1: The propensity score distribution using histogram and density plot among children born to Aboriginal mothers, Northern Territory, Australia, 2002-2017.

Figure S2: The propensity score distribution using histogram and density plot among children born to non-Aboriginal mothers, Northern Territory, Australia, 2002-2017.

Table S8: Standardised mean difference and variance ratio to evaluate covariate balance among children born to Aboriginal and non-Aboriginal mothers, Northern Territory, Australia, 2002-2017.

| **Variables** | **Children born to Aboriginal mothers** | | **Children born to non-Aboriginal mothers** | |
| --- | --- | --- | --- | --- |
|  | **Standardised mean difference** | **Variance ratio** | **Standardised mean difference** | **Variance ratio** |
| History of MHrH | 0.0147 | 1.0613 | 0.0132 | 1.1215 |
| Maternal age | 0.1399 | 0.8742 | -0.0269 | 0.9368 |
| Gestational age | 0.0149 | 0.9731 | -0.0660 | 1.2171 |
| Parity | 0.0723 | 1.1575 | 0.0024 | 0.8921 |
| Antenatal care visit | -0.0548 | 1.0249 | -0.0024 | 1.0051 |
| Smoking during pregnancy | 0.0235 | 1.0887 | -0.0030 | 1.0488 |
| Alcohol during pregnancy | 0.0355 | 0.9611 | 0.0488 | 1.0931 |
| Sex | -0.1226 | 0.9703 | 0.0364 | 1.0022 |
| Neonatal admission to special unit | 0.0264 | 0.9703 | -0.0379 | 1.0714 |
| Method of delivery | -0.0253 | 0.9187 | 0.0061 | 1.0451 |
| Birth weight | 0.0142 | 0.9730 | -0.0590 | 1.2189 |
| Urinary tract infection | 0.0521 | 1.1923 | n.a. | n.a. |
| Renal disease | 0.0527 | 1.3187 | n.a. | n.a. |
| Pre-existing hypertension | 0.0113 | 1.0844 | n.a. | n.a. |
| Pre-existing diabetes mellitus | 0.0273 | 1.1643 | n.a. | n.a. |
| Cardiac disease | -0.0860 | 0.6915 | n.a. | n.a. |
| Gestational disease | 0.0009 | 1.0024 | n.a. | n.a. |
| Intrauterine growth retardation | -0.0136 | 0.9469 | n.a. | n.a. |
| Fetal distress | -0.0961 | 0.7939 | 0.0025 | 1.0080 |
| Meconium-stained liquor | -0.0231 | 0.9507 | -0.0170 | 0.9578 |
| Obstructed labour | -0.0098 | 0.9644 | -0.0161 | 0.9591 |
| Postpartum haemorrhage | -0.0749 | 0.8731 | 0.0107 | 1.0365 |
| Antepartum haemorrhage | 0.0094 | 1.0715 | n.a. | n.a. |
| Pre-eclampsia | -0.0312 | 0.8130 | n.a. | n.a. |
| Anaemia | -0.0339 | 0.9086 | n.a. | n.a. |

**n.a. = variables not used due to low cell count values.**

**Table S9: Percentage of the specific mental health conditions among children born to Aboriginal and non-Aboriginal mothers in the Northern Territory, Australia, 2002-2017.**

| **Specific mental health conditions** | **Children from Aboriginal mothers (n, %)** | **Children from non-Aboriginal mothers (n, %)** |
| --- | --- | --- |
| Severe mental health conditions | 39 (0.19%) | 45 (0.12%) |
| Common mental health conditions | 92 (0.44%) | 180 (0.47%) |
| Personality disorders | n.p. | 10 (0.03%) |
| Other adult-onset conditions | 55 (0.27%) | 92 (0.24%) |
| Other childhood-onset conditions | 12 (0.06%) | n.p. |
| Substance-related mental-health conditions | 277 (1.34%) | 62 (0.16%) |

**n.p. = results not presented due to low cell count values**
